# Feasibility of "CopeSmart": A Telemental Health App for Adolescents

**DOI:** 10.2196/mental.4370

**Published:** 2015-08-10

**Authors:** Rachel Kenny, Barbara Dooley, Amanda Fitzgerald

**Affiliations:** ^1^ School of Psychology University College Dublin Dublin 4 Ireland

**Keywords:** adolescents, emotional self-monitoring, feasibility, mobile apps, positive mental health

## Abstract

**Background:**

Early intervention is important in order to improve mental health outcomes for young people. Given the recent rise in mobile phone ownership among adolescents, an innovative means of delivering such intervention is through the use of mobile phone applications (apps).

**Objective:**

The aim of this study was to evaluate the feasibility of “CopeSmart”, a telemental health app developed to foster positive mental health in adolescents through emotional self-monitoring and the promotion of positive coping strategies.

**Methods:**

Forty-three adolescents (88% female) aged 15-17 years downloaded the app and used it over a one-week period. They then completed self-report questionnaires containing both open-ended and closed-ended questions about their experiences of using the app. The app itself captured data related to user engagement.

**Results:**

On average participants engaged with the app on 4 of the 7 days within the intervention period. Feedback from users was reasonably positive, with 70% of participants reporting that they would use the app again and 70% reporting that they would recommend it to a friend. Thematic analysis of qualitative data identified themes pertaining to users’ experiences of the app, which were both positive (eg, easy to use, attractive layout, emotional self-monitoring, helpful information, notifications, unique) and negative (eg, content issues, did not make user feel better, mood rating issues, password entry, interface issues, engagement issues, technical fixes).

**Conclusions:**

Overall findings suggest that telemental health apps have potential as a feasible medium for promoting positive mental health, with the majority of young people identifying such technologies as at least somewhat useful and displaying a moderate level of engagement with them. Future research should aim to evaluate the efficacy of such technologies as tools for improving mental health outcomes in young people.

## Introduction

Adolescence has consistently been identified as a critical period for the development of mental health difficulties [1-3], with mental health disorders affecting approximately one in five adolescents internationally [4,5]. There is increasing evidence to suggest the importance of early intervention in reducing severity of symptoms, preventing onset of further mental health difficulties, and generally improving mental health outcomes for young people [6]. A potentially cost-effective means of delivering such interventions is to do so remotely through the use of mobile technologies, a practice which falls under the umbrella of telemedicine and is known as “telemental health” [7].

Recent years have seen dramatic increases in smartphone ownership, with approximately 69-84% of adolescents in the developed world now owning smartphones [8-10]. This type of technology is a promising tool for the widespread delivery of telemental health interventions to young people. Because adolescents are familiar with how to use mobile phones (as they use them on a daily basis for texting, taking photos, playing games etc), they are unlikely to have much difficulty in adapting to the intervention interface [11]. Interventions delivered via mobile phones facilitate frequent engagement as users go about their daily lives [12,13] and afford users the opportunity to apply the skills and behaviors promoted by the intervention directly in real time to their real life experiences [14]. They can provide almost constant support to users, in comparison to interventions that can only be accessed at specific times or in specific locations (eg, during visits to a therapist’s office) [14]. Furthermore, as it is not unusual to see young people interacting with their mobile phones in public, they provide a discrete and confidential means of intervention delivery [11].

Over the coming years, it is predicted that the use of mobile technologies in mental health contexts will continue to rapidly increase [15]. Indeed, there are already a plethora of health care mobile apps available to adolescents [15], with over 3000 mental health apps accessible for download between the Android, Apple, and Microsoft app stores [16]. These apps have been developed by a wide range of individuals and organizations and are generally available free or at a small cost [17]. Some are targeted towards the assessment and treatment of specific mental health disorders, whereas others are focused on the more broad promotion of positive mental health [18] (ie, by fostering general well-being and resilience in individuals). Many of these apps are based on self-monitoring principles, where users rate how they are feeling on a day to day basis and are able to track their moods and emotions over time, for example “Moody Me” [19] and “Mood Panda” [20]. Some apps also provide advice on dealing with differing mental health concerns such as “Overcoming Depression” [21] and “Mental Health WATS” [22]. However, the problem with almost all of these apps is that they are not subject to regulatory assessments or empirical evaluations before they are made available to the general public and very few are supported by research indicting their effectiveness and usability [15,17,23]. This lack of research may be a consequence of most apps reaching consumers directly, without going through traditional medical gatekeepers [18]; however, it means that the quality of the content of these apps cannot be verified. Thus, the information these apps provide could be inaccurate or misleading, which at best may not be useful to users or at worst may cause them psychological harm [17]. There is a growing demand for studies evaluating the feasibility and efficacy of rigorously designed, evidence-based apps before they are made publically available through online platforms [7,15,23,24].

As rigorously designed evaluations often require the investment of significant resources, not all interventions can be tested for efficacy and effectiveness. Feasibility studies play an important role in identifying potentially efficacious interventions and ensuring they are prioritized for comprehensive evaluation [25]. Feasibility studies achieve this by exploring factors such as the acceptability of an intervention to participants, adherence and drop-out rates, and aspects of the intervention that may be altered to improve participants’ engagement [26], and are considered a crucial first step prior to the conduction of larger scale efficacy evaluations [27,28].

Whittaker et al [29], for example, explored the acceptability of cognitive behavioral therapy text-messages delivered twice daily to adolescents over nine weeks. They found that three-quarters of participants found the information useful and 90% would recommend the intervention to a friend. Similarly, Reid et al [30] explored usage patterns of a simple diary-based self-monitoring mobile app for adolescents and found engagement with the app was high, with nearly three-quarters of participants completing 80% or more of possible diary entries over the one-week intervention period. Findings from these studies are promising and suggest more complex app-based mental health interventions may be similarly feasible for use with adolescents.

The objective of the present study was to evaluate the feasibility of a telemental health app, which aimed to foster positive mental health, for use by adolescents on their personal mobile phones. This study expands on previous research [29,30] by capturing detailed qualitative and quantitative feedback from adolescents pertaining to their satisfaction with, perceived usefulness of, and overall engagement with a telemental health app.

## Methods

### App Design

The app in the present study was designed and programed for use on android and iOS mobile devices, following focus group consultation with adolescents in relation to their needs from mental health mobile technologies [31]. The aim of the app was to foster positive mental health via two key mechanisms: (1) emotional self-monitoring, and (2) the promotion of positive coping strategies. Increasing awareness of emotions is a process frequently used in therapeutic contexts [32] and periodically monitoring one’s emotions (ie, engaging in emotional self-monitoring) has been shown to facilitate increases in emotional self-awareness and improvement in mental health outcomes [33,34]. There is also a growing awareness in research literature that the ways in which young people cope with stressors may be significantly correlated with their mental health outcomes [1,35,36], and educating adolescents about coping strategies has been linked to increased use of positive coping strategies and improved mental health [37-39].

**Figure 1:**
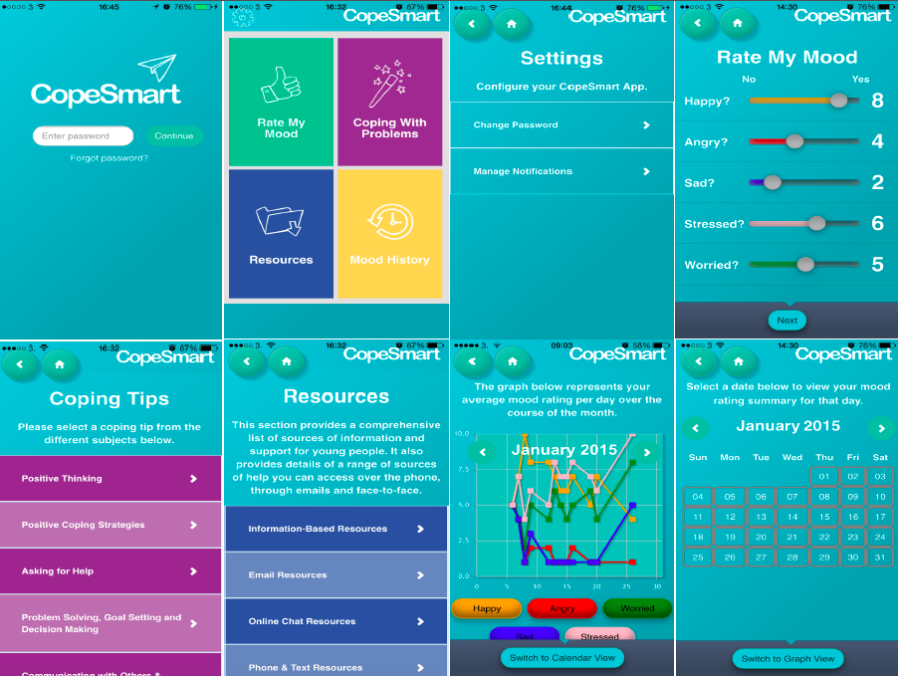
CopeSmart app screenshots.

The app was branded “CopeSmart” and contains five main sections (see Figure 1 for sample screenshots). *Section 1* (Settings) gives users the option to set and change their password for accessing the app and to manage their notification settings. By default the app is set to send reminder notifications to users to engage with it at 8pm each evening, as typically by this time of the day most adolescents are finished with school and after-school activities. *Section 2* (Rate My Mood) allows users engage in emotional self-monitoring by rating how happy, angry, sad, stressed or worried they feel on a scale of 1-10. *Section 3* of the app (Coping Tips) contains a series of “tips” for coping with problems. These tips were devised based on the “Think Positively” school-based course for developing positive coping strategies in adolescents [40]. One of these tips is randomly selected to display each time a user completes a mood rating and in Section 2, all tips are available for users to browse at their leisure. *Section 4* (Resources) provides users with contact details for a list of mental health support services. These include online resources (eg ReachOut.com), phone and text resources (eg, the Samaritans helpline) and face-to-face resources (eg, their school guidance counsellor or GP). In *Section 5* (Mood History), users are able to look back at their mood ratings over time, in either a calendar or graph format.

### Feasibility Evaluation

#### Participants

Participants were 43 adolescents (88% female) aged 15-17 years with a mean of 16.0 years (SD 0.724), in possession of Android or iOS mobile phone devices.

#### Procedure

Approval for this study was granted by the Human Research Ethics Committee in University College Dublin. Initially, emails were sent to six schools in the East of the Republic of Ireland detailing the nature of the study. Four of these schools agreed to participate (three urban, one rural). None of the schools were considered socially or economically disadvantaged under nationally established governmental criteria [41]. Students in fourth and fifth year (equivalent to 10^th^ & 11^th^ Grade in the United States) were invited to participate. Students attending a one-day program hosted by University College Dublin that allows fifth year students to experience aspects of university life, were also invited to participate. The coordinator of this program was contacted and permission was granted to recruit participants from this group. Potential participants were provided with information sheets and consent/assent forms for themselves and their parents. Participation was entirely voluntary and participants were not offered any monetary incentive to take part in the research.

Initially, over 250 young people were approached and of these 215 returned parental consent and were eligible to participate. Of these eligible participants, 114 did not download the app, primarily due to unforeseen technical delays which meant the app was unavailable on iOS or a number of Android devices for a period of time. Consequently, many eligible participants from the first two schools and the one-day university program were unable to download the app. Of the 101 participants who downloaded the app, 43% completed follow-up questionnaires and these individuals made up the final sample used in the present study. The low follow-up rate was mainly due to restrictions on when the researchers could access schools, which meant that a significant number of students were absent on the day of questionnaire completion due to either exam prep or involvement in extracurricular activities.

The 43 participants who comprised the final sample downloaded and installed the app on their personal mobile phones during school hours. Participants were asked to engage with the app and rate their mood on a regular basis over a brief one-week period (as is common in feasibility studies which generally have shorter follow-up periods than larger scale trials [25]). After one week participants completed a follow-up self-report questionnaire containing closed-ended and open-ended questions pertaining to their experiences of using the app (see Multimedia Appendix 1). The app itself captured data relating to user engagement, which was sent to a back-end server when the device came into contact with a wireless Internet connection.

### Data Analysis

Descriptive statistics were used to present quantitative data. Thematic analysis [42] was used to analyze qualitative feedback. A set of initial themes (ie, patterned responses) was identified from participants’ responses to open-ended questions. These themes were revised and refined, and a coding frame listing the final themes was established (see Table 2 for list of themes). A second researcher independently applied this coding frame to the data, and a Cohen’s kappa inter-rater reliability coefficient of .76 was computed [43]. This is considered to be a good level of reliability [44].

## Results

### Questionnaire Responses

Table 1 illustrates participants’ usefulness ratings of the app and its main subcomponents. In addition, 79% of participants reported that they liked the interface layout, 7% disliked it, and 14% neither liked nor disliked it. The majority of participants (93%) found the app easy to use, whereas 7% (n=3) reported some minor technical difficulties with logging on. Approximately 70% of participants would use the app in the future, 74% felt that other young people would use it, and 70% would recommend it to a friend.

**Table 1:** Participants’ usefulness ratings.

	Very useful	Somewhat useful	Not useful
	n (%)	n (%)	n (%)
Overall	3 (7%)	31 (72%)	9 (21%)
Mood rating^a^	15 (35%)	18 (42%)	7 (16%)
Coping tips^b^	8 (19%)	23 (54%)	10 (23%)
Resources^c^	5 (12%)	20 (40%)	17 (40%)

^a^missing data for mood rating n=3; ^b^missing data for coping tips n=2; ^c^missing data for resources n=1

Table 2 outlines the themes identified from participants’ responses to open-ended questions, across two categories: (1) aspects of the app they liked and reasons why they or other young people would use the app in the future or recommend it to a friend; and (2) aspects of the app they did not like and what changes they would recommend to improve the app.

**Table 2:** Qualitative feedback from participants.

Aspects of the app participants liked/reasons they or other young people would use it in future or recommend it to a friend.		
Theme	Example	n^a^
Easy to use	“*It is free and easy to use”*	9
Attractive layout	“*I liked the appearance of the app”*	6
Mood rating was generally helpful	“*I liked the way you can rate your mood”*	17
Mood rating helped increase emotional self-awareness	“*This app helps me to stop and take a second to listen to how I actually feel”*	16
Mood rating was useful as alternate emotional outlet	“*A friend might find it easier to share stuff with an app rather than friends”*	2
Information on app was helpful	“*I liked the helpful tips”*	15
Notifications were useful	“*It reminded me to rate my mood”*	5
Uniqueness of app	“*It’s the only app I’ve found like it”*	3
Needs more information	“*More coping advice”*	8
Needs less information	“*There was too much to read”*	2
Needs to be more personalized	“*The info that came up wasn’t really related to me”*	4
Information provided was not useful	“*I didn’t find it all that helpful”*	6
Did not make user feel better	“*It didn’t make me feel any better”*	7
Needs more moods to rate	“*I wish there were more emotions”*	6
Mood charts were confusing	“*Graphs should be easier to read”*	5
Should have option to input text	“*Have space for a small diary”*	4
Password entry is annoying	“*No password every time”*	6
Interface issues	“*More colors and fancy text”*	9
More activities	”*If it had something else, such as games…I’d be more inclined to use it”*	6
Uninterested in app/do not need it	“*Not my thing really”*	7
Technical fixes	“*Get the chat resources in just one click”*	4

^a^n=number of participants who identified this theme

### Usage Data

**Figure 2:**
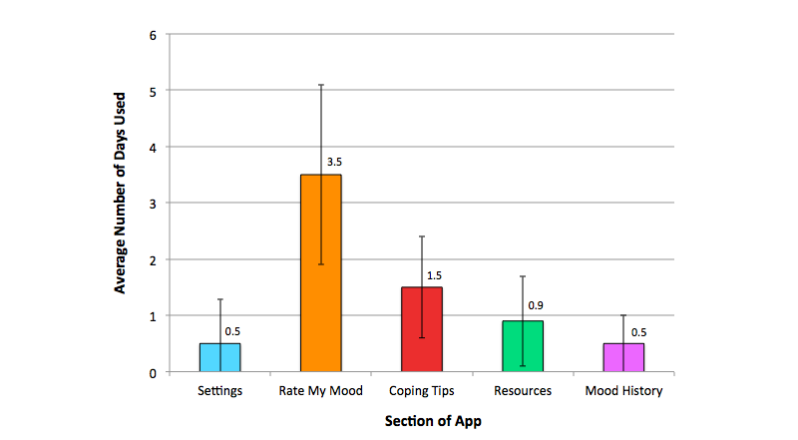
Average number of days within the usage period on which participants engaged with different sections of the app (within 1 standard deviation).

Usage data were obtained from 30 participants, as it was not possible to obtain usage data from all participants due to differing security settings across mobile devices. On average, participants engaged with the app on 4 of the days within the 7-day usage period (SD 1.75). As illustrated in Figure 2, participants most frequently interacted with Section 2 of the app (Rate My Mood), with participants on average using this section on 3.5 days during the intervention period. The variance in terms of the average number of days each section was used was high among participants, as illustrated by the standard deviation bars presented in the chart.

**Figure 3:**
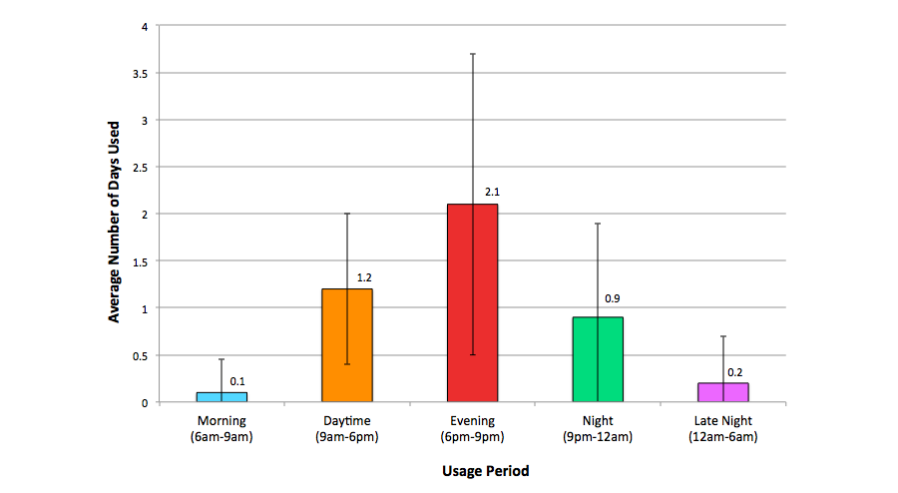
Average number of days within the usage period on which participants engaged with the app during specific time periods (within 1 standard deviation).

Figure 3 displays the times at which users most frequently engaged with the app. Evening (6pm-9pm) was the most popular time for participants to use the app, followed by daytime (9am-6pm) and night (9pm-12am), although again variance in usage time was high, as illustrated by the standard deviation bars in the chart.

## Discussion

### Principal Findings

Overall, participants’ responses to the app were reasonably positive, with 79% of participants reporting they found it at least somewhat useful and 93% reporting that they found it easy to use. Nearly three-quarters of participants reported that they would use the app in the future and would recommend it to a friend. User engagement levels were moderate, with participants on average engaging with the app on 4 of the 7 days within the intervention period. These findings suggest this type of app has potential as a feasible means of promoting positive youth mental health.

Participants identified “Rate My Mood” as the most useful section, and most frequently engaged with this component. As in Reid et al’s study [30], where young people’s primary responses to the self-monitoring app were that they liked how it helped them to understand themselves, users in the present study liked that the app helped them increase awareness of their emotions. This is a positive finding given that previous research has found mobile self-monitoring tools have the potential to increase emotional awareness and improve mental health outcomes [33,34]. Interestingly, the “Mood History” section was used very little, suggesting the mere act of stopping to momentarily think about one’s mood may be more salient for adolescents than retrospectively tracking mood over time. However, it is also possible that the time frame for app usage in the present study was not sufficient to build enough mood data for participants to be interested in viewing their mood history over time. As in previous research on young people’s needs using telemental health apps [31], participants suggested the app could be helpful as an emotional outlet for people who are not comfortable talking about their feelings, and that users should be able to input text into the app containing details about their emotions. This is a useful finding, as writing about emotional experiences can be linked to improvements in well-being [45].

Participants’ engagement levels with the “Coping Tips” and “Resources” components of the app were low, and 23% and 40% of participants respectively reported that they did not find these sections helpful. Their main reasons were that they felt they did not need this information or that it did not help them to feel better. This corresponds to the primary reasons given by participants in Whittaker et al’s text message-based intervention as to why they did not find the intervention content useful [29], and emphasises the importance of ensuring adolescents are involved in the design of telemental health interventions to ensure the information they provide is relevant and helpful to young people [46]. However, engagement with the coping content may be underestimated by the data captured in the present study, as a coping tip was displayed to users every time they rated their mood using Section 2. Furthermore, nearly three-quarters of participants reported that they found the Coping Tips at least somewhat useful, with over half of participants reporting that they found them very useful, suggesting this content was helpful for the majority of participants.

Six participants reported that having to enter a password every time they accessed the app acted as a barrier to engagement. As privacy has been identified as essential for adolescents in these types of interventions [11,31], a challenge faced in future development will be to reconcile this conflict between ease of engagement and protecting privacy. Participants found the notifications useful for encouraging engagement and evening was the most popular time for app usage, possibly linked to the fact that reminder notifications were set by default to 8pm. Morning and late night were the least popular times, in contrast to Reid et al’s study where engagement was evenly distributed across time points [30]. Future research should explore the use of reminder notifications at different times to assess when users are most likely to engage with these technologies.

The high levels of participant variability in terms of engagement with different sections of the app and the times at which the app was used, indicates that young people engaged with the app in different ways and to different extents. Similarly, there was variability and some level of contradiction in terms of qualitative feedback obtained; for example, some participants reported that they wanted more coping information and some reported that they wanted less. A challenge faced by future researchers will be how to reconcile such contradictory feedback and a key focus going forward should be to identify characteristics associated with different levels of app engagement and specific users’ needs (ie, how do needs differ for different types of users, what types of users are most likely to engage with such technologies, and under what circumstances). This will help to identify patterns of engagement with, and individual user needs from such technologies, so that telemental health interventions can be tailored and personalized to target specific types of users. Indeed, this is in line with the comments of four participants in the present study who specifically reported a desire for more personalized content in the app.

### Limitations and Strengths

A limitation of this study was the gender imbalance of the sample, which meant statistical analysis could not be conducted to explore gender differences in participants’ responses. As research suggests males are less likely to show interest in technology-based mental health interventions [29], and indeed are less likely to participate in scientific research in general [47], exploring gender differences in engagement levels should be a priority for future research. Furthermore, the sample size in the present study was reasonably small due to numerous logistical challenges faced during the data collection procedure. While a small sample size is often used in preliminary feasibility studies [25], future research should aim to evaluate these types of technologies in larger samples of adolescents using standardized quality assessment scales such as the recently developed Mobile Application Rating Scale [48]. Strengths of the current study include the capture of real-time data to explore app engagement, which has an advantage over retrospective self-report data that can be subject to recall bias whereby participants overestimate the frequency of their behaviors [49]. Furthermore, capturing both quantitative and qualitative data allowed for an in-depth exploration of participants’ experiences of app usage.

### Conclusions

Overall findings suggest that telemental health apps have potential as a feasible medium for promoting positive youth mental health, with the majority of young people identifying such technologies as at least somewhat useful and displaying a moderate level of engagement with them. Future research should aim to evaluate the effectiveness of such technologies as tools for improving mental health outcomes, using rigorously controlled research designs. This piece of research was conducted as part of a larger study, the next phase of which will be to evaluate the efficacy of the app as a tool for fostering positive mental health in adolescents via emotional self-monitoring and the promotion of positive coping strategies. This will be done using a large sample of young people and with a randomized controlled design methodology.

## Appendix

Multimedia Appendix 1 Self-Report Questionnaire.
