# Supplementary material for: Feasibility of "CopeSmart": A Telemental Health App for Adolescents
Source: JMIR Ment Health. 2015 Aug 10;2(3):e22. doi: 10.2196/mental.4370 (PMC4705022; doi:10.2196/mental.4370)
Supplement: Multimedia Appendix 1 [file mental_v2i3e22_app1.pdf]

Questionnaire

INTRODUCTION

Thank you for taking part in this research. Please answer as many questions as you can. There are **no** right or wrong answers & we would like you to answer as honestly as possible.

This is a **confidential** questionnaire and your answers will be **anonymous.** This means no-one will know what you have written and we will not pass on personal information to your parents/guardians or anyone at your school. The only exception to this is if you reveal that you may harm yourself or others. If this happens, the research team must let your school know that one of their students is at risk. However, because your responses are anonymous you will not be identified personally.

In order to link up your questionnaire to your app data we would like you to enter your anonymous identification code below. The code should follow this format:

Box 1: Second Letter of your First Name (e.g. if your name is MARY; enter A in Box 1)
Box 2: Month of your birthday, the last 3 letters (e.g. if you were born in APRIL; enter RIL in Box 2)
Box 3: Date of your birthday (e.g. if you were born on the 8th of the month; enter 08 in Box 3)
Box 4: Year you were born in (e.g. if you were born in 1995, enter 95 in Box 4)
Box 5: First Letter of your Surname (e.g. if your surname is SMITH; enter S into Box 5)

(i.e. using the examples given above the code generated would be: ARIL0895S)

If you are unclear about this, please ask the researcher for help.

YOUR CODE:

**Box 1 (second letter of your first name)**	
**Box 2 (month your birthday is in, the last 3 letters)**	
**Box 3 (date of your birthday)**	
**Box 4 (year you were born in)**	
**Box 5 (first letter of your surname**	

Question 1: What is your gender?

Male	○
Female	○

Question 2: What age are you? ____

Question 3: What school do you go to? ______________________________________

Question 4: How useful did you find the app overall?

I didn't find it useful at all	○
I found it somewhat useful	○
I found it very useful	○

Question 5: How much did you find each of the following useful?

**Mood Rating?**	
I didn't find it useful at all	○
I found it somewhat useful	○
I found it very useful	○
**Coping Information?**	
I didn't find it useful at all	○
I found it somewhat useful	○
I found it very useful	○
**Resources?**	
I didn't find them useful at all	○
I found them somewhat useful	○
I found them very useful	○

Question 6: How much did you like the appearance/layout of the app?

I didn't like it at all	○
I somewhat disliked it	○
I neither liked nor disliked it	○
I liked it	○
I liked it very much	○

Question 7: How would you improve the appearance/layout of the app?

____

____

____

____

____

____

Question 8: How easy/difficult was the app to use?

Very difficult to use	○
Somewhat difficult to use	○
Easy to use	○
Very easy to use	○

Question 9 (a) Did you have any difficulties using any parts of the app?

Yes	○
No	○

Question 9 (b) If yes, please explain what these difficulties were.

____

____

____

____

____

____

Question 10: What did you like about the app?

Question 11: What did you dislike about the app?

Question 12: How would you improve the app?

Question 13: (a) Would you use the app in the future?

Yes	○
No	○

Question 13: (b) Why/Why not?

Question 14: (a) Do you think other young people would use the app?

Yes	○
No	○

Question 14: (b) Why/Why not?

Question 15: (a) Would you recommend the app to a friend?

Yes	○
No	○

Question 15: (b) Why/Why not?

Question 16: Any other comments?
